# Caring Behaviours in Nursing: A Bibliometric Analysis of Global Research Trends and Knowledge Structure

**DOI:** 10.1111/scs.70318

**Published:** 2026-10-01

**Authors:** İlknur Özkan, Seçil Taylan

**Affiliations:** ^1^ İnternal Medicine Nursing Department Kumluca Faculty of Health Sciences, Akdeniz University Kumluca Türkiye; ^2^ Surgical Nursing Department Kumluca Faculty of Health Sciences, Akdeniz University Kumluca Türkiye

**Keywords:** bibliometric analysis, caring behaviours, nursing

## Abstract

**Background:**

Caring behaviours constitute one of the most visible ways in which “caring” is translated into everyday nursing practice, yet the field has become increasingly heterogeneous across settings, outcomes, and conceptual traditions. A field‐level map is therefore needed to clarify how the literature has evolved and where it is currently concentrating.

**Objective:**

This study aimed to bibliometrically map the literature on caring behaviours in nursing, focusing on global research trends, thematic development, collaboration networks, citation performance, and the field's intellectual structure.

**Methods:**

A bibliometric analysis was conducted on publications indexed in the Web of Science Core Collection between 1987 and 2025, following the SALSA framework. Analyses included performance indicators (publication growth; most productive journals and countries), Author Keyword‐based frequency, trend‐topic, co‐occurrence, and thematic analyses following synonym merging and spelling harmonization, co‐authorship network analysis (collaboration patterns), citation analyses, and co‐citation mapping to examine the field's intellectual structure.

**Results:**

A total of 351 documents from 158 sources (1987–2025) were included. The literature demonstrated steady growth, accelerating after 2016. Core source outlets included *BMC Nursing*, *Journal of Nursing Administration*, and *Perspectives in Psychiatric Care*. The USA, Iran, China, and Turkey were among the most productive countries, while international co‐authorship remained limited (14.25%). Thematic signals suggested a shift from earlier emphases on patient satisfaction toward a more recent focus on nursing education, empathy, emotional intelligence, and, most recently, compassion fatigue. The thematic map positioned caring behaviours within the basic‐themes quadrant, whereas work environment and related humanistic‐care concepts showed greater thematic development. Foundational influences remained anchored in Watson and Swanson, and measurement‐oriented works (notably Wu and Wolf) functioned as key intellectual bridges.

**Conclusions:**

Caring behaviours research appears to be a maturing but still expanding field with strong theoretical and measurement “anchors,” yet comparatively constrained cross‐national collaboration. Future work may benefit from more internationally coordinated programmes and greater attention to cross‐cultural validation of measurement tools and to the emotional, organizational, and digitally mediated contexts in which caring behaviours are enacted.

## 1 Introduction

Caring has long been positioned as a defining moral and practical foundation of nursing, shaping both the profession's ethical commitments and its enactment in everyday clinical work [1, 2, 3]. Within nursing theory, caring is conceptualized not as an adjunct to technical competence but as an orienting stance that structures the nurse–person relationship and guides the manner in which care is delivered [4, 5, 6]. From this perspective, the quality of nursing care is expressed not only through accuracy and task completion but also through relational processes that affirm personhood, preserve dignity, and respond to individual needs. Caring theories have therefore provided a conceptual vocabulary for articulating what patients and nurses often recognize within clinical encounters: That care is experienced and evaluated through how nurses attend, communicate, and remain present, even amidst organizational constraints [7, 8, 9].

In this theoretical and practice context, the construct of caring behaviours has gained prominence as a means of operationalizing caring in observable and measurable terms [10, 11]. Caring behaviours can be understood as behavioural expressions through which nurses convey respect, attentiveness, supportive presence, and responsiveness features that enable patients to interpret nursing care as genuinely person‐centred rather than primarily procedural [11, 12]. In this sense, caring behaviours provide an empirical link between the relational ideals of caring and their expression in everyday nursing practice. The construct is also relevant to professional nursing identity in so far as it reflects the enactment of nursing values in routine practice and offers a bridge between normative theoretical commitments and empirically tractable phenomena [1, 2, 9].

Over the past decades, research on caring behaviours has expanded across a wide range of clinical settings, patient populations and cultural contexts, generating a substantial and increasingly heterogeneous evidence base. However, as the literature diversifies, synthesizing its development through conventional narrative approaches becomes more challenging [13, 14]. Patterns of research attention, thematic evolution, scholarly connectivity and intellectual foundations may be difficult to appraise without a field‐level overview. In the absence of such mapping, it remains unclear how the evidence base has evolved over time, where research efforts have concentrated, which conceptual and methodological traditions have shaped the field, and which areas remain comparatively underexamined [13, 14, 15]. This limits the capacity to position new studies strategically, to identify knowledge gaps systematically and to align future research with both theoretical debates and practice priorities.

Bibliometric methods provide a systematic approach for mapping the structure and evolution of a research field by examining publication growth, influential sources, co‐authorship and collaboration patterns, and thematic and intellectual linkages [13, 15]. Accordingly, the present study aims to conduct a bibliometric mapping of the nursing literature on caring behaviours, situated within the SALSA (Search, Appraisal, Synthesis and Analysis) framework [16]. By offering an integrated overview of the field's development and structure, this study seeks to support conceptually grounded and strategically oriented agendas for future research and to inform implications for nursing education and clinical practice.

## 2 Method

This study was designed as a bibliometric analysis conducted in accordance with the BIBLIO checklist for reporting bibliometric studies of biomedical literature [17] (File S1). In addition, the study followed the SALSA (Search, Appraisal, Synthesis and Analysis) framework to ensure a systematic and transparent process of literature identification and analysis [16]. The SALSA framework was selected because it provides a structured, transparent, and reproducible approach for searching, appraising, synthesizing, and analysing the literature, which aligns with the aim of systematically mapping the development and structure of the caring behaviours literature. In this bibliometric study, the Appraisal stage referred to the application of predefined eligibility criteria rather than to a formal methodological quality assessment of individual studies.

### 2.1 Aim

The aim of this study was to provide a comprehensive bibliometric evaluation of the literature on caring behaviours in nursing, focusing on global research trends, thematic development, collaboration networks, citation performance, and the intellectual structure of the field.

### 2.2 Research Questions

This study addresses the following research questions:

1. What are the publication trends and growth patterns in the literature on caring behaviours in nursing?
2. Which journals, authors, and countries are the most productive and influential within the field?
3. What is the structure of scientific collaboration at the author and country levels?
4. Which publications constitute the intellectual foundation of the field based on citation and co‐citation analyses?
5. What are the most frequent Author Keywords, and how have research themes evolved over time?
6. How is the conceptual structure of the field characterised according to network and thematic map analyses?

### 2.3 Database and Study Collection

The study data were retrieved from the Web of Science Core Collection (WoSCC) database. The search included the Science Citation Index Expanded (SCIE), Social Sciences Citation Index (SSCI), Arts & Humanities Citation Index (AHCI), and Emerging Sources Citation Index (ESCI). WoSCC was preferred due to its high‐quality indexing standards, comprehensive citation coverage, and long‐term archival consistency. Its standardised bibliographic and citation metadata also facilitate reproducible bibliometric analyses. Using a single database helped maintain consistency in record structure, indexing conventions, and citation information throughout the analysis.

The search strategy was constructed using the Topic (TS) field in Web of Science, which includes title, abstract, Author Keywords, and Keywords Plus. The following query was applied:

TS = ((“caring behaviour*” OR “caring behaviour*” OR “nursing care behaviour*” OR “nursing care behaviour*” OR “care behaviour*”) AND nurs*) NOT TS = (“self‐care behaviour*” OR “self‐care behaviour*” OR “self care behaviour*” OR “self care behaviour*” OR “health behaviour*” OR “health behaviour*” OR “patient behaviour*” OR “patient behaviour*” OR “risk behaviour*” OR “risk behaviour*”).

The search was conducted in February 2026, and all records available in the selected WoSCC indexes on the search date were initially retrieved for screening. Because 2026 represented an incomplete publication year, all records assigned to 2026 were excluded before the final bibliometric analyses. Accordingly, the final analytical period covered 1987–2025. The exclusion of “self‐care behaviours” was applied to prevent conceptual drift toward patient self‐management and health behaviour research, ensuring that the dataset remained focused on caring behaviours performed by nurses rather than patients' self‐care behaviours. Importantly, retrieval through the Topic field was used only to identify potentially relevant records; inclusion in the final dataset was determined through subsequent eligibility screening rather than by Topic‐field retrieval alone. Although Keywords Plus contributed to record retrieval through the TS field, keyword‐based conceptual analyses were conducted using Author Keywords (DE), as described in the analysis section.

### 2.4 Eligibility Criteria

Publications were included if they were written in English, indexed in the Web of Science Core Collection, and directly related to caring behaviours in nursing. Studies were considered directly related if they focused on caring behaviours performed by nurses or nursing students, perceptions of nurses' caring behaviours, or measurement and evaluation of caring behaviours in nursing practice. Studies in which caring behaviours constituted a substantive study concept, outcome, or measurement construct were considered eligible; records in which caring appeared only as an incidental or indirect component of a broader concept were excluded. Both original research articles and review articles, including Early Access records classified as these document types, were included in the analysis to provide a comprehensive overview of the intellectual and conceptual development of the field. No year limitation was applied during the search process to capture the entire scope of the literature available in the database.

Publications were excluded if they were not written in English, were duplicate records, or lacked essential bibliographic information required for bibliometric analysis, such as title, author name, publication year, source title, or citation information. The absence of a DOI or abstract alone was not considered a reason for exclusion. Editorials, letters, conference abstracts, book chapters, corrections, and retracted papers were also excluded. Studies were also excluded if they focused primarily on patient self‐care behaviours, health behaviours, risk behaviours, patient behaviours, or general care‐related topics that did not specifically address caring behaviours performed by nurses. Studies from disciplines outside nursing were excluded when caring behaviours in nursing did not constitute the primary focus of the publication.

### 2.5 Selection Process

The SALSA framework (Search, Appraisal, Synthesis, and Analysis) was employed to ensure a systematic, transparent, and reproducible selection process (Figure 1). The search was conducted in the Web of Science Core Collection database without applying any year restriction to capture the full scope of the literature on caring behaviours in nursing.

**FIGURE 1:**
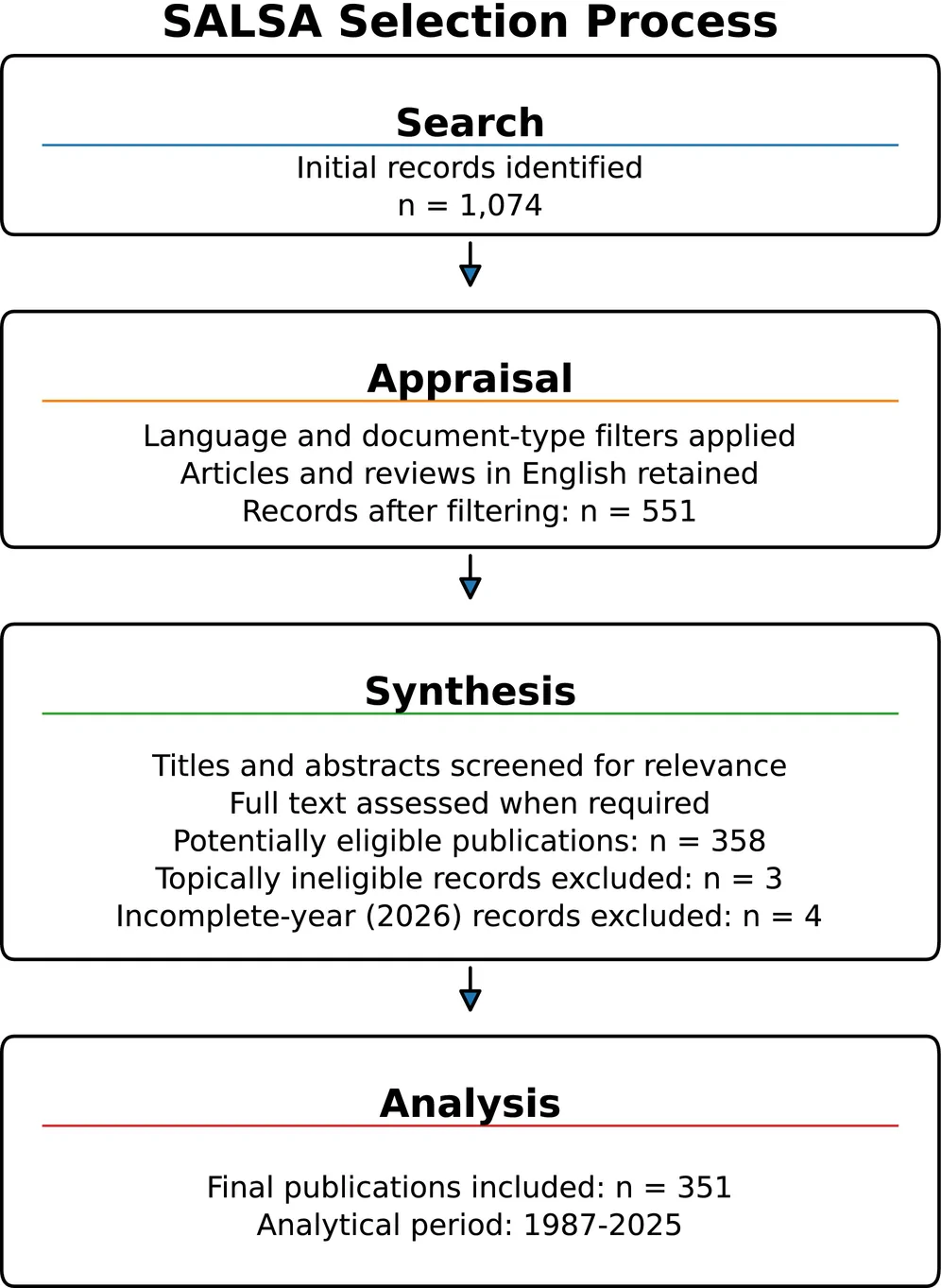
SALSA flow diagram of the study selection and bibliometric analysis process.

The initial search yielded 1074 records. During the appraisal stage, document type and language filters were applied in accordance with the predefined eligibility criteria. Only articles and review articles written in English, including records with Early Access status, were retained, reducing the dataset to 551 records.

In the synthesis stage, titles and abstracts were systematically screened to assess their relevance to caring behaviours in nursing. Publications not directly related to the research theme, duplicate records, and studies with incomplete bibliographic information were excluded. When necessary, full‐text screening was conducted to confirm eligibility. This screening initially identified 358 potentially eligible publications. A final eligibility verification was then performed to ensure that caring behaviours in nursing constituted a substantive focus of each record. Three records that did not meet the topical eligibility criteria were excluded. In addition, four records assigned to the incomplete 2026 publication year were excluded. Following the completion of all screening procedures, 351 publications met the inclusion criteria and were included in the final bibliometric analysis.

### 2.6 Data Analysis

Bibliometric data analysis was conducted using R (version 4.3.1) through RStudio. The Bibliometrix package and its Biblioshiny interface were employed to perform performance analysis and science mapping procedures [15]. Before conducting the analyses, a data cleaning procedure was performed using Biblioshiny to improve conceptual consistency across the dataset. Author Keywords (DE) were manually reviewed and harmonised before keyword‐based analyses. A predefined synonym list was used to merge singular and plural forms, spelling variations, punctuation variants, and semantically equivalent expressions (e.g., “nurse/nurses,” “caring behaviour/caring behaviours,” and “nursing student/nursing students”) (File S2, Table S1). However, conceptually related but analytically distinct terms were not automatically merged. For example, “nurses” and “nursing” were retained separately because they represent the professional group and the disciplinary field, respectively; similarly, “caring behaviours” and “caring” were retained separately because the former refers to a measurable construct, whereas the latter reflects a broader theoretical concept. “Nursing care” was also retained separately from “caring behaviours” because it represents a broader concept of care delivery. Measurement‐related terms such as reliability, validity, validation, psychometric properties, and instrument development were likewise retained as distinct concepts (File S2, Table S2). No additional removal list was applied; preprocessing was limited to synonym merging, spelling correction, punctuationstandardisation, and singular/plural harmonization to minimise researcher‐driven alteration of the conceptual structure. The complete preprocessing and harmonization decisions are provided in File S2, Table S1 and Table S2.

In the first stage, descriptive bibliometric indicators were calculated, including the total number of publications, annual growth rate, average citations per document, average document age, and total number of references. The temporal distribution of publications was examined to identify changes in scientific production over time. Performance analysis was conducted to evaluate source and author productivity. Bradford's Law was applied to identify core journals and characterise the distribution of publications across source zones, while Lotka's Law was used to examine the distribution of author productivity. Global and local citation analyses were performed to identify influential publications and authors within the field. To examine the social structure of the literature, co‐authorship analyses were conducted at both author and country levels, allowing collaboration patterns, international partnerships, and structurally central actors to be identified.

The conceptual structure of the field was examined exclusively using Author Keywords (DE). Although Keywords Plus were available in the WoSCC records and are reported descriptively as part of the bibliographic dataset, they were not used in keyword frequency, trend‐topic, co‐occurrence, centrality, or thematic‐map analyses. Author Keywords were selected because they more directly represent the concepts emphasised by the authors of the original publications. Keyword frequency and co‐occurrence analyses were performed to identify dominant concepts and their relationships. A minimum co‐occurrence threshold of ≥ 5 was applied to retain terms with sufficient frequency for network analysis. Thematic mapping based on Callon's centrality and density measures was used to classify themes as motor, basic, niche, or emerging/declining. Trend‐topic analysis was also conducted to examine changes in thematic emphasis over time.

Finally, network centrality measures, including betweenness centrality, closeness centrality, and PageRank, were calculated to identify structurally influential nodes within the Author Keyword co‐occurrence network, author and country collaboration networks, and the co‐citation network.

### 2.7 Ethical Considerations

This study is based exclusively on published bibliometric data retrieved from a publicly accessible database. It does not involve human participants, animals, or personal data. Therefore, ethical approval from an institutional review board was not required.

## 3 Results

### 3.1 General Characteristics of the Bibliometric Data

The final dataset comprised 351 publications on caring behaviours in nursing published between 1987 and 2025 across 158 sources. The annual scientific production remained relatively limited during the early years of the field, followed by a marked increase after 2016. Publication output accelerated particularly from 2019 onward and reached its highest level in 2025 (Figure 2). The average document age was 8.41 years, while the mean number of citations per document was 13.52. The publications collectively cited 8975 references. The dataset contained 647 Author Keywords and 363 Keywords Plus terms. A total of 1185 authors contributed to the literature, with 30 single‐authored publications and an average of 3.97 co‐authors per document. International co‐authorship accounted for 14.25% of the publications, suggesting that although collaborative authorship was common, cross‐national collaboration remained comparatively limited (Table 1).

**FIGURE 2:**
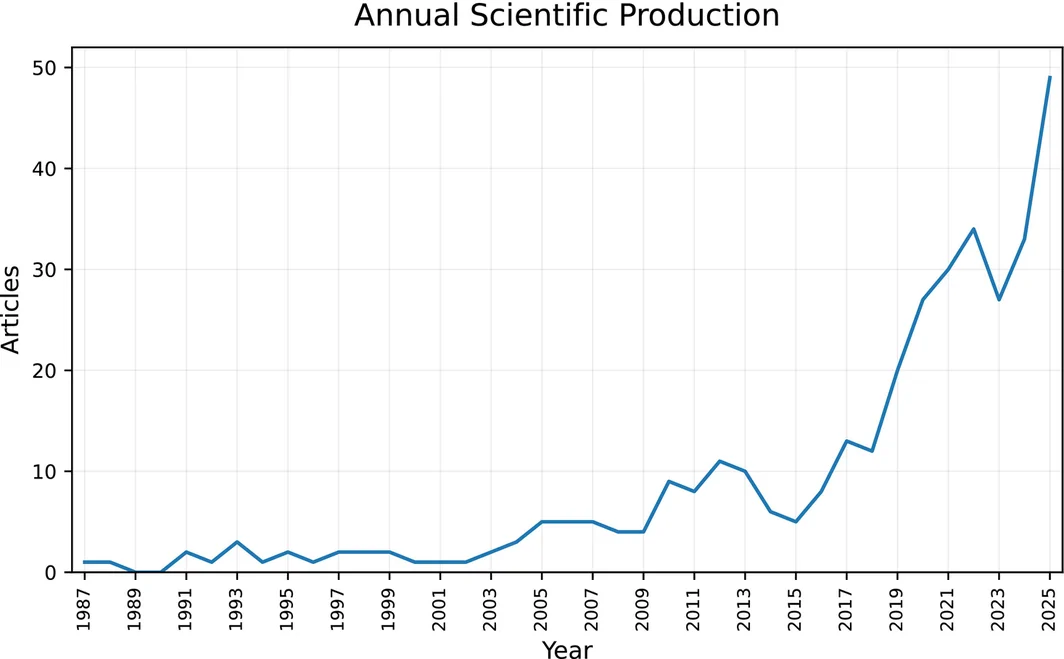
Annual scientific production of publications on caring behaviours in nursing, 1987–2025.

**TABLE 1:** Main bibliometric characteristics of the dataset.

Category	Indicator	Value
Main Information About Data	Timespan	1987–2025
	Sources (Journals, Books, etc.)	158
	Documents	351
	Annual Growth Rate (%)	10.78
	Document Average Age	8.41
	Average Citations per Document	13.52
	Total References	8975
Document Contents	Keywords Plus (ID)	363
	Author's Keywords (DE)	647
Authors	Total Authors	1185
	Authors of Single‐Authored Documents	28
Authors Collaboration	Single‐Authored Documents	30
	Co‐Authors per Document	3.97
	International Co‐authorships (%)	14.25

### 3.2 Most Relevant Sources

The analysis of the most relevant sources shows that *BMC Nursing* was the leading journal with 28 publications. *Journal of Nursing Administration* and *Perspectives in Psychiatric Care* followed with 13 publications each, while *Nurse Education in Practice* contributed 10 publications. *Nurse Education Today* published 7 articles, and *Journal of Advanced Nursing*, *Journal of Clinical Nursing*, and *Nursing Ethics* each contributed 6 publications. *Cancer Nursing* and *Enfermería Clínica* each accounted for 5 publications (Figure S1). These findings indicate that research on caring behaviours is concentrated primarily within a relatively small group of nursing journals, while also extending across clinical, educational, ethical, and speciality‐oriented outlets. Bradford's Law analysis further confirms the presence of a distinct core group of sources, led by *BMC Nursing*, *Journal of Nursing Administration*, *Perspectives in Psychiatric Care*, and *Nurse Education in Practice* (Figure S2). Beyond this core, publications are progressively dispersed across a larger number of journals, reflecting both concentration and disciplinary breadth within the field.

### 3.3 Author Productivity and Local Citation Impact

Boz, I. was the most productive author with seven publications, followed by Lee‐Hsieh, J. and Papastavrou, E. with six publications each. Karlou, C., Patiraki, E., Tsangari, H., and Turton, M. A. each contributed five publications, while Duffy, J. R., Efstathiou, G., and Kuo, C. L. each authored four (Figure S3). In terms of local citation impact, Larrabee, J. H., Putman, H. P., and Wu, Y. ranked first with 71 local citations each, followed by Papastavrou, E. with 51. Karlou, C. and Patiraki, E. each received 38 local citations, Tsangari, H. received 34, Efstathiou, G. and Merkouris, A. received 33 each, and Papathanasiou, I. V. received 32 (Figure S4). Lotka's Law analysis showed a strongly skewed productivity pattern, with most authors contributing only one publication and progressively fewer authors contributing multiple papers. This distribution suggests that the field is supported by a small group of recurrent contributors within a considerably larger population of occasional authors (Figure S5).

### 3.4 Corresponding Authors' Countries and Collaboration Pattern

The United States ranked first with 85 publications (79 SCP; 6 MCP), followed by Iran with 45 publications (41 SCP; 4 MCP) and China with 38 publications (33 SCP; 5 MCP). Turkey contributed 32 publications, of which 30 were single‐country publications and 2 involved international collaboration. Indonesia accounted for 19 publications (17 SCP; 2 MCP). Cyprus showed the highest proportion of multiple‐country publications among the leading contributors, with 5 of its 8 publications involving international collaboration. Sweden contributed 8 publications (7 SCP; 1 MCP), while Ethiopia produced 7 publications (5 SCP; 2 MCP). Italy and Malaysia each contributed 6 publications, with Malaysia showing a relatively high MCP proportion (3 of 6 publications). Overall, single‐country publications predominated across most countries, although several smaller contributors, particularly Cyprus, Malaysia, Egypt, Italy, and Ethiopia, demonstrated comparatively stronger international collaboration patterns (Figure S6, Table S3).

### 3.5 Most Globally and Locally Cited Documents

Table 2 presents the most influential documents according to local, global, and normalised citation indicators. Wu (2006) was the most locally influential publication, with 71 local citations and 166 global citations. Sarafis (2016) received the highest number of global citations (*n* = 235) and also showed the highest normalised global citation score (5.38). Labrague (2017) recorded the highest normalised local citation score (6.80), indicating particularly strong influence within the caring behaviours literature relative to its publication year. Omari (2013) had the highest local‐to‐global citation ratio (53.84%), suggesting that a substantial proportion of its citation impact was concentrated within the field itself. Overall, the citation patterns indicate that some publications have achieved broad international visibility, whereas others have exerted a more concentrated influence within the intellectual structure of caring behaviours research in nursing (Table 2).

**TABLE 2:** Most influential documents ranked by local, global, and normalised citation indicators.

Document	Year	Local citations	Global citations	LC/GC ratio (%)	Normalized local citations	Normalized global citations
Wu Y, 2006, Nurs Res	2006	71	166	42.77	4.17	3.56
Cronin SN, 1988, Heart Lung	1988	24	85	28.23	1	1
Labrague LJ, 2017, Int J Nurs Knowl	2017	22	58	37.93	6.80	4.71
Sarafis P, BMC	2016	17	235	7.23	4.68	5.38
Palese A, 2011, J Nurs Scholarship	2011	16	59	27.11	4.57	2.74
Khademian Z, 2008, J Adv Nurs	2008	15	49	30.61	2.60	1.86
Burtson PL, 2010, J Adv Nurs	2010	15	100	15	2.75	3.50
Wade GH, 2006, J Nurs Educ	2006	14	41	34.14	0.82	0.87
Murphy F, 2009, Nurs Educ Today	2009	14	77	18.18	2.54	2.31
Omari FH, 2013, J Clin Nurs	2013	14	26	53.84	4.82	2.09

*Note:* Normalised citation indicators adjust citation counts according to publication year to facilitate fairer comparisons across documents published in different periods.

Abbreviations: GC, Global Citations; LC, Local Citations.

### 3.6 Author Keywords: Frequency and Trend Analysis

Following keyword harmonization, caring behaviours were the most frequent Author Keyword (*n* = 124), followed by nurses (*n* = 65), caring (*n* = 62), nursing students (*n* = 48), nursing (*n* = 45), and nursing care (*n* = 38). Other frequently occurring terms included nursing education (*n* = 21), patient satisfaction (*n* = 16), perceptions (*n* = 15), and empathy (*n* = 13) (Figure 3).

**FIGURE 3:**
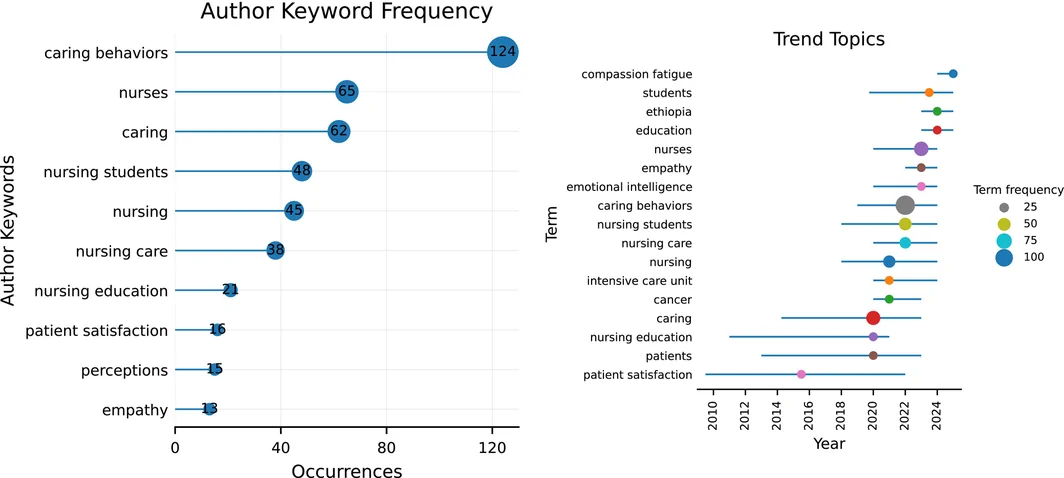
Author keyword frequency and trend topics.

The trend‐topic analysis indicates a gradual change in thematic emphasis over time. Patient satisfaction represents one of the earlier prominent topics, with its median occurrence around 2016. Caring, patients, and nursing education became more visible around 2020, followed by nursing, intensive care unit, and cancer around 2021. Caring behaviours, nursing students, and nursing care were particularly prominent around 2022. More recently, emotional intelligence, empathy, and nurses became visible around 2023, while education, Ethiopia, and students were concentrated around 2024. Compassion fatigue appeared as the most recent trend, centred around 2025 (Figure 3). Overall, the temporal pattern suggests a movement from earlier patient‐oriented outcomes, particularly patient satisfaction, toward greater attention to nursing education, nurses' relational and emotional competencies, and the psychological demands associated with sustaining caring practice.

### 3.7 Author Keyword Co‐Occurrence Network Analysis

The co‐occurrence network was constructed exclusively using Author Keywords (DE) after synonym merging and spelling harmonization. The network comprised six clusters, although most of the conceptual connectivity was concentrated in two major groups (Figure 4). Cluster 1 was centred on caring behaviours, which showed the highest betweenness centrality (608.15) and PageRank (0.196), followed by nurses (betweenness = 227.53; PageRank = 0.100) and caring (betweenness = 102.64). This cluster also included patient satisfaction, perceptions, emotional intelligence, ethical sensitivity, burnout, compassion fatigue, work environment, and several clinical‐context terms, reflecting the clinical, relational, and professional dimensions of caring.

**FIGURE 4:**
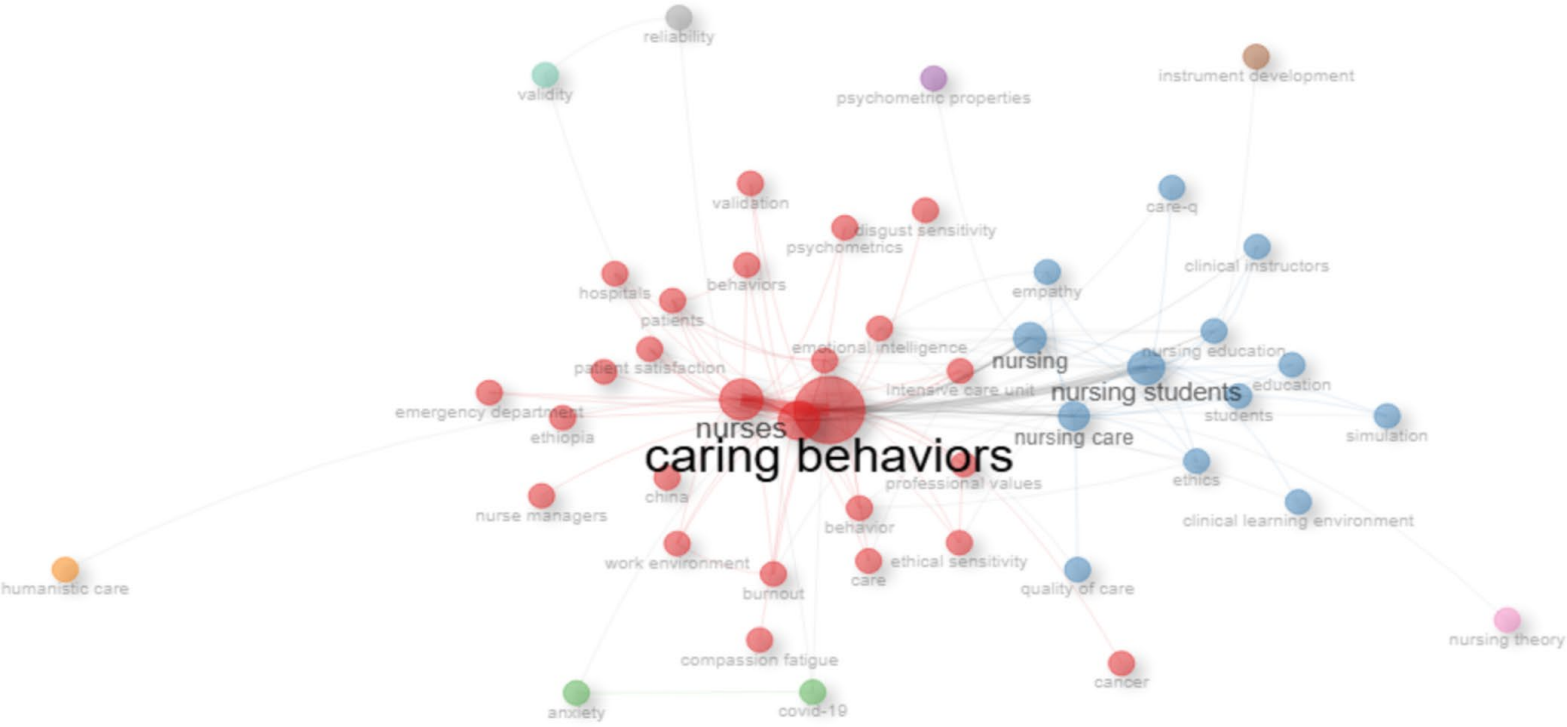
Author keyword co‐occurrence network.

Cluster 2 was organized around nursing care, nursing students, nursing, and nursing education. Nursing care showed the highest betweenness within this cluster (76.30), followed by nursing students (56.79), nursing (54.19), and nursing education (45.47). The cluster primarily represented educational and competency‐related dimensions, including empathy, simulation, clinical instructors, and the clinical learning environment. Smaller peripheral clusters were organized around COVID‐19 and anxiety, psychometric properties, humanistic care, and instrument development. Overall, caring behaviours occupied the strongest bridging position among the Author Keywords, connecting clinical, educational, emotional, and methodological strands of the literature (Figure 4). Detailed centrality indicators for the Author Keyword network are provided in Table S4.

### 3.8 Thematic Map Analysis

The thematic map was generated using Author Keywords (DE) and classified themes according to Callon centrality and density. The map shows that work environment, together with closely associated terms such as humanistic care and China, occupies the motor‐theme quadrant, indicating a relatively well‐developed thematic structure with stronger connections to the broader field. In contrast, the largest thematic group, centred on behaviours, is positioned within the basic‐themes quadrant (centrality = 1.71; density = 23.87). This group also includes closely connected terms such as nurses, nursing students, nursing education, clinical instructors, and simulation, suggesting that these concepts form a broad and highly relevant foundation of the literature but remain comparatively less internally developed (Figure 5, Table S5).

**FIGURE 5:**
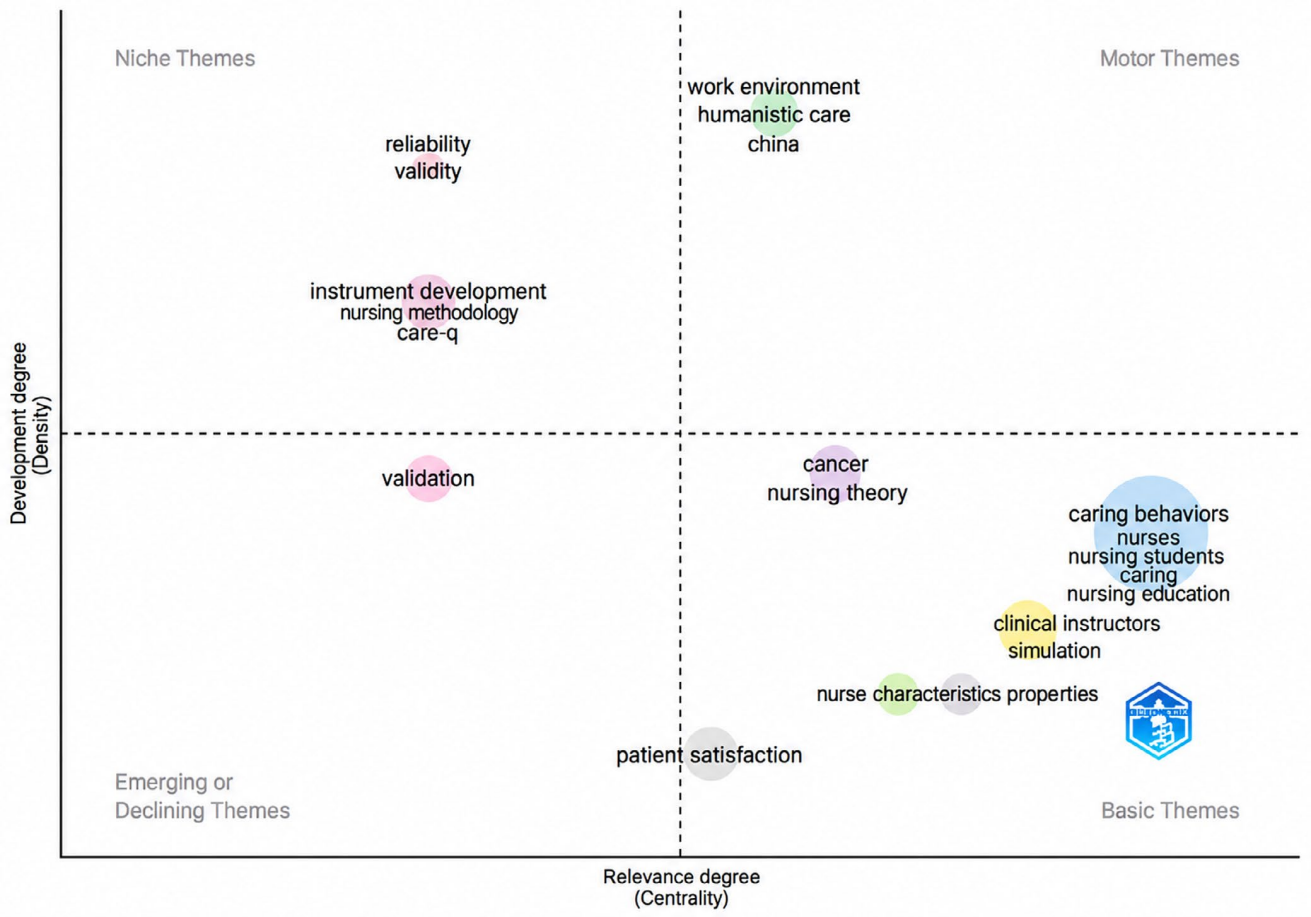
Thematic map based on author keywords.

Several specialised themes are located in the niche‐theme quadrant. These include reliability and validity, as well as instrument development and related measurement‐oriented terms, reflecting internally developed but relatively peripheral lines of inquiry. Validation appears in the emerging or declining quadrant, characterised by low centrality and low density. Psychometric properties also occupy a comparatively peripheral position, with low density despite some degree of centrality (centrality = 0.17; density = 20.00). Patient satisfaction remains within the basic‐theme area but shows particularly low density (centrality = 0.05; density = 6.25), indicating continued relevance to the field despite limited internal thematic development. Overall, the thematic structure suggests that caring behaviours and nursing education‐related concepts constitute the broad conceptual base of the field, while work‐environment and humanistic‐care themes show greater thematic development and measurement‐related themes remain more specialised or peripheral (Figure 5). Detailed Callon centrality, density, rank, and cluster‐frequency indicators are provided in Table S5.

### 3.9 Author Collaboration Network

The author collaboration network comprised 11 distinct clusters, indicating a fragmented structure characterised by several relatively independent research groups. The largest and most established group was Cluster 10, centred on Papastavrou, E., and included Karlou, C., Patiraki, E., Tsangari, H., Efstathiou, G., Merkouris, A., Jarosova, D., Leino‐Kilpi, H., and Suhonen, R. A separate collaboration group was formed around Lee‐Hsieh, J., in Cluster 9, together with Turton, M. A., Kuo, C. L., and Tseng, H. F (Figure 6).

**FIGURE 6:**
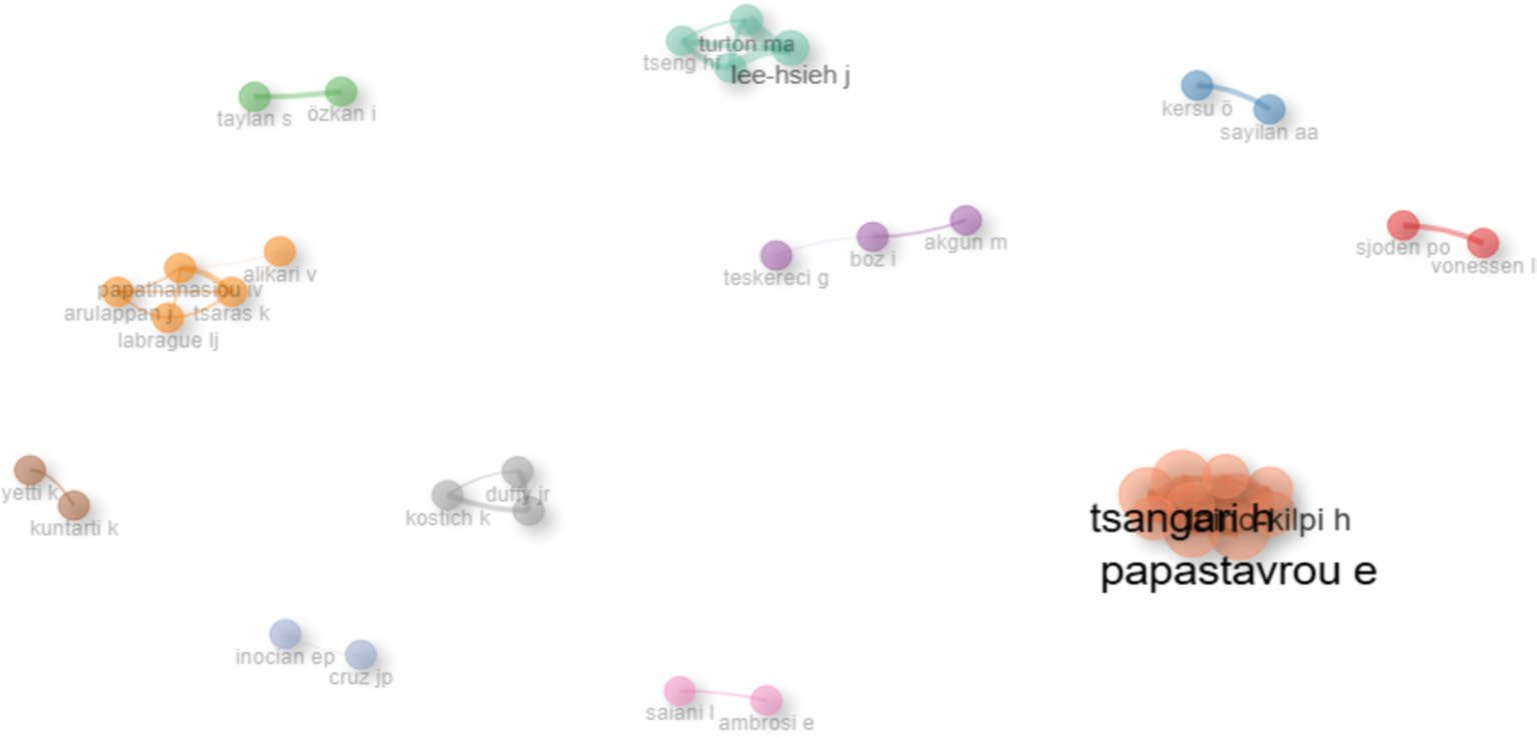
Author co‐authorship network.

Betweenness centrality values were generally low across the network. Boz, I., in Cluster 4 and Papathanasiou, I. V. and Tsaras, K., in Cluster 5, had the highest betweenness values (1.00), suggesting limited bridging roles between otherwise weakly connected author groups. Smaller and more isolated partnerships were observed in Cluster 1 (Sjoden, P. O. and Vonessen, L.), Cluster 3 (Taylan, S. and Özkan, I.), and Cluster 11 (Cruz, J. P. and Inocian, E. P.). Overall, the network suggests that collaboration in caring behaviours research is organized around several semi‐independent teams rather than a densely interconnected author community (Figure 6). Detailed centrality measures are provided in Table S6.

### 3.10 Country Collaboration Network

The country collaboration network comprised eight clusters, reflecting geographically and institutionally differentiated patterns of international cooperation (Figure 7). The **USA**, located in Cluster 3, showed the highest betweenness centrality (327.70) and PageRank (0.109), confirming its dominant bridging role within the global network. This cluster also included Turkey, Jordan, Oman, Malawi, Nigeria, India, and Ireland. Cluster 6 represented a European collaboration structure comprising Italy, Greece, Cyprus, Finland, the Czech Republic, and Hungary; within this group, Italy (betweenness = 133.49) and Greece (77.90) occupied particularly prominent bridging positions. Cluster 1 linked Saudi Arabia, the Philippines, Egypt, Kazakhstan, and Bahrain, with Saudi Arabia showing substantial betweenness centrality (92.17). Cluster 5 included China, Indonesia, Malaysia, and Thailand, reflecting an Asian collaboration pattern, while Cluster 7 connected Iran, Canada, the United Kingdom, and Pakistan. Sweden and Ethiopia formed Cluster 4, and Japan and Australia constituted a small isolated pair in Cluster 8. Overall, the network indicates that international collaboration is structured around the USA as the principal global connector, alongside several regionally concentrated collaboration groups (Figure 7). Detailed country‐level centrality indicators are provided in Table S7.

**FIGURE 7:**
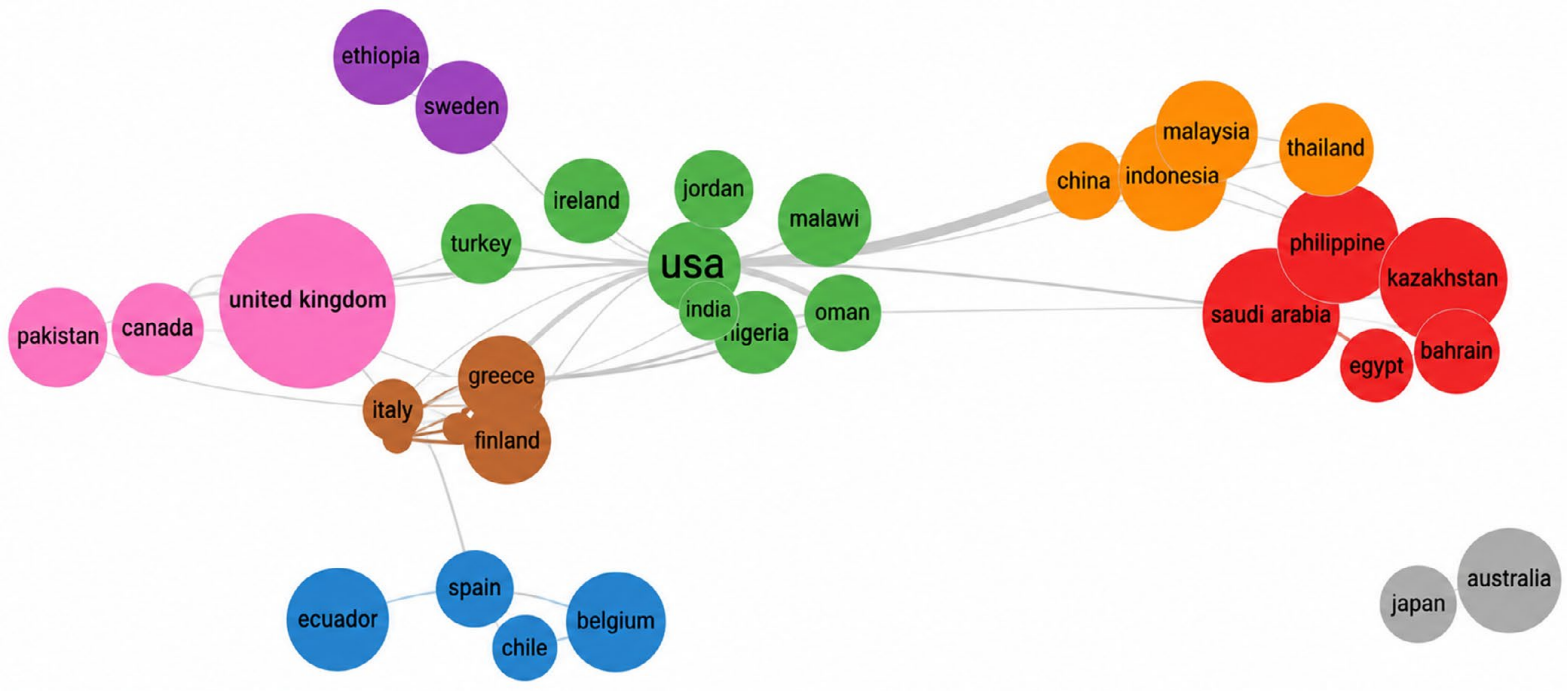
Country collaboration network.

### 3.11 Co‐Citation Network

The co‐citation network was organized into two major intellectual clusters. Wu (2006) occupied the most prominent bridging position in the network, with the highest betweenness centrality (373.44) and PageRank (0.066), followed by Wolf (1994) (betweenness = 268.40; PageRank = 0.054). Cluster 1 brought together foundational theoretical and measurement‐oriented references, including Watson, Swanson, Wolf, Cronin, Papastavrou, and Palese, reflecting the conceptual and psychometric foundations of caring behaviours research. Within this cluster, Papastavrou (2011) and Watson (2008) also showed notable bridging positions, linking theoretical perspectives with empirical applications (Figure 8, Table S8).

**FIGURE 8:**
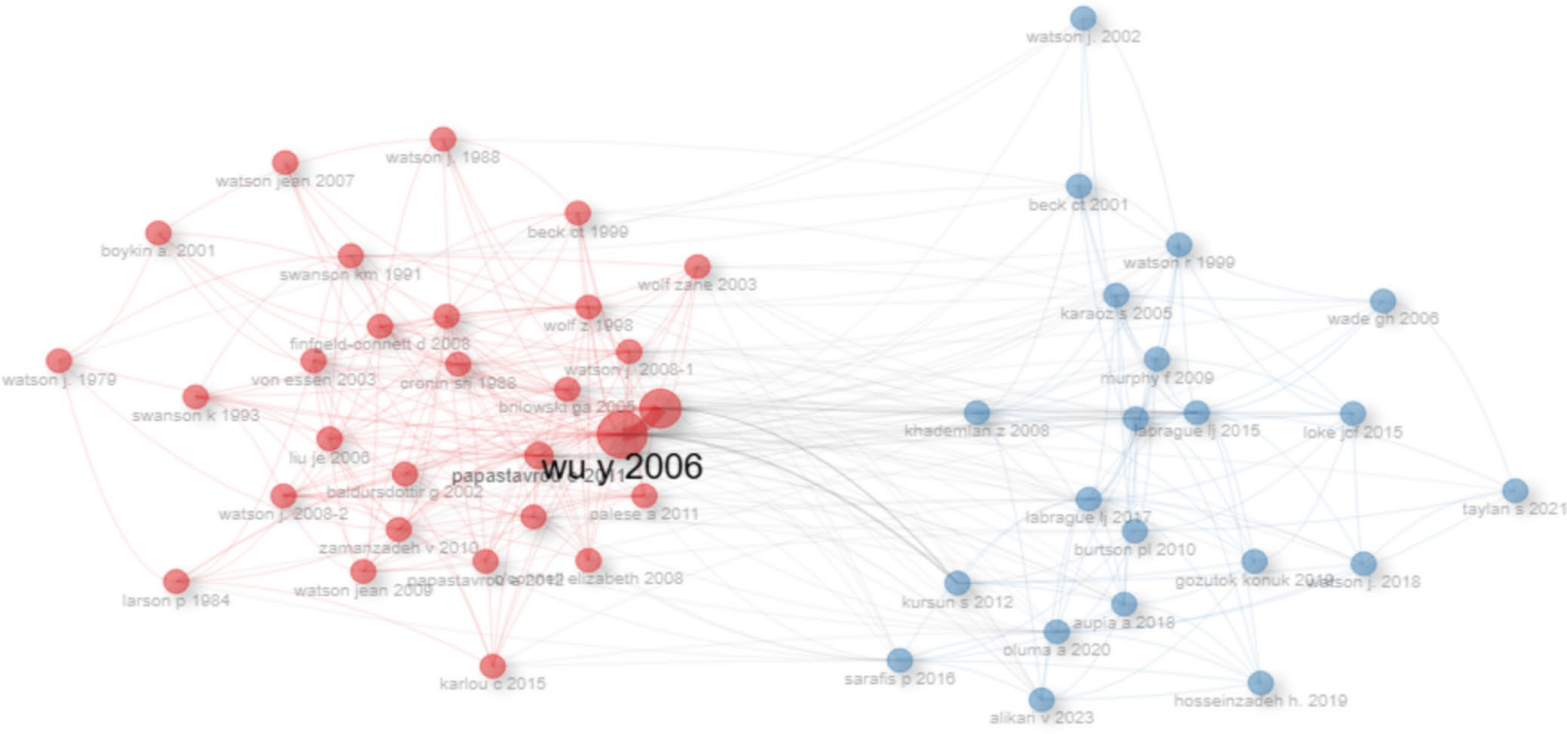
Co‐citation network of referenced documents.

Cluster 2 was composed mainly of more recent empirical studies examining caring behaviours in relation to nursing education, occupational stress, patient outcomes, and clinical practice. Within this cluster, Khademlan (2008) (betweenness = 233.55), Sarafis (2016) (118.08), Oluma (2020) (106.24), and Labrague (2015) (67.51) occupied comparatively influential positions. Overall, the network indicates that the intellectual structure of the field is shaped by a close interaction between longstanding theoretical and measurement foundations and a more recent body of applied empirical research. Wu (2006) remains the principal bridging reference connecting these intellectual strands (Figure 8). Detailed co‐citation centrality indicators are provided in Table S8.

## 4 Discussion

This bibliometric analysis indicates that the literature on caring behaviours in nursing expanded substantially over the study period, with a marked acceleration after 2016 and further growth in the most recent years (Table 1, Figure 2). The fact that 351 publications are distributed across 158 sources suggests that caring behaviours have not remained confined to a single niche; rather, they have developed into a research area that cuts across clinical practice, nursing education, management, and measurement and evaluation. At the same time, the relatively modest rate of international co‐authorship (14.25%) implies that, despite the field's expanding international presence, collaborative activity still tends to cluster within regional networks. This pattern is consistent with the country collaboration analysis, in which the USA functions as the principal global connector while several European, Asian, and Middle Eastern partnerships remain organized within relatively distinct regional clusters.

Our results point to a change in emphasis in the caring behaviours literature. Earlier studies often treated caring mainly through its visible consequences for patients, most notably satisfaction with nursing care [18]. The updated trend analysis supports this temporal pattern, with patient satisfaction emerging as one of the earlier prominent topics, whereas caring behaviours, nursing students, and nursing care became more visible around 2022, followed by empathy, emotional intelligence, and nurses in more recent years. Compassion fatigue emerged as the most recent trend, centred around 2025. This shift suggests that caring is increasingly being discussed as something nurses must be able to sustain, not only something patients receive. Evidence from education aligns with this view. In student samples, empathy tends to move alongside caring‐related competencies, highlighting the developmental side of caring [19, 20, 21]. Although ethical sensitivity did not emerge among the most recent trend topics, its position within the Author Keyword co‐occurrence network indicates that it remains closely connected with caring behaviours and professional values. Ethical principles and ethical sensitivity show meaningful links with students' caring behaviours [22, 23, 24].

Work conditions also matter. Notably, work environment emerged within the motor‐theme quadrant of the updated thematic map, suggesting that organizational conditions have become both relatively well developed and increasingly connected to the broader conceptual structure of the field. Studies consistently report that occupational stress and job stress are associated with weaker caring behaviours [25, 26, 27]. When this pressure becomes ongoing, the effects may reach patients as well; evidence syntheses link nurse burnout with poorer quality of care and lower patient satisfaction [28, 29, 30]. Compassion fatigue further sharpens this picture [31, 32, 33]. Typically discussed within professional quality‐of‐life frameworks [34, 35, 36], it is now examined more directly alongside caring behaviours, including in oncology and intensive care settings where emotional demands are particularly high [31, 37]. Professional identity may also be part of the explanation, helping to clarify how workplace conditions translate into day‐to‐day caring practice [38, 39, 40]. Taken together, these patterns suggest a gradual broadening of the field from an earlier emphasis on patient‐reported outcomes toward a more layered understanding of caring that also encompasses nurses' emotional resources, professional development, and the organizational conditions under which caring is sustained.

Co‐citation analysis indicates that the caring behaviours literature still rests on a strong intellectual backbone. The central position of established measurement tools such as those by Wu (2006) and Wolf (1994), alongside the theoretical frameworks of Watson and Swanson within the co‐citation network, suggests conceptual continuity in the field and indicates that its intellectual structure continues to be shaped by long‐standing theoretical and measurement traditions [5, 6, 11, 41]. This continuity is particularly evident in the updated co‐citation network, where Wu (2006) occupies the strongest bridging position, and Wolf (1994) also shows high centrality, linking foundational measurement work with subsequent empirical research. However, the updated thematic map presents a more nuanced picture of measurement‐related research. Rather than “psychometric properties” occupying the emerging/declining quadrant, it remains comparatively peripheral and shows relatively low thematic density, while “validation” is located in the emerging/declining quadrant. In contrast, themes related to reliability, validity, and instrument development appear as more specialised niche themes. This pattern suggests not that measurement and evaluation studies have lost their importance, but rather that methodological work has developed along relatively specialised lines and remains less integrated with the broader thematic core of caring behaviours research. Therefore, instead of describing existing measurement tools as “inadequate,” it may be more appropriate to emphasise that, while these tools remain valuable and widely used, they may need to be re‐examined in changing care environments in terms of content validity and measurement invariance and, where necessary, supported with additional dimensions that capture communication, accessibility, and a sense of “presence” in digitally mediated or remote care encounters [42, 43, 44]. Such re‐examination would be especially relevant as caring interactions increasingly extend beyond conventional face‐to‐face settings. This approach may contribute to developing an evaluation framework that preserves the field's classical foundations while measuring contemporary care dynamics more sensitively.

The relatively low rate of international co‐authorship (14.25%) and the predominance of single‐country publications (SCP) suggest that knowledge generation in caring behaviours research is often shaped within national or regional contexts. The updated country collaboration network reinforces this pattern: Although the USA occupies the strongest bridging position in the network, international collaboration is distributed across several relatively distinct European, Asian, Middle Eastern, and other regional clusters rather than forming a densely integrated global network. Such a structure may partly reflect geographic proximity and established academic partnerships, although bibliometric data alone cannot determine the organizational or funding mechanisms underlying these collaborations. However, interpreting limited international collaboration only as a “weakness” risks overlooking an important feature of caring behaviours. Caring behaviours are closely tied to culturally sensitive factors such as the language used in patient–nurse communication, how privacy is maintained, how professional roles are understood, and what patients and nurses expect from “good care.” Studies comparing nurses' and patients' perceptions of caring behaviours across clinical settings indicate that these perceptions may not always coincide and can be influenced by contextual characteristics [45, 46]. This makes international collaborative work especially valuable, but it also makes it methodologically more demanding. In multi‐country studies, it is not enough to simply collect data from different samples; researchers also need evidence that the measurement tools capture the same construct in comparable ways across cultural and linguistic settings. For this reason, the goal of developing a “universal model of care” may require not a single one‐size‐fits‐all template, but a framework that identifies a common conceptual core while allowing culturally responsive expressions of caring to vary across settings. From this perspective, limited international collaboration does not completely prevent the development of such a framework. Instead, it may slow the accumulation of comparative evidence needed to distinguish which dimensions of caring remain stable across contexts and which are culturally contingent. Future research would therefore benefit from coordinated multi‐country studies using harmonised protocols and explicitly testing cross‐cultural measurement equivalence. Using shared measurement tools can facilitate such comparisons, provided that their validity is not simply assumed across settings. In addition, existing scales may need to be re‐examined as caring increasingly extends into remote and digitally mediated encounters, where communication, accessibility, and relational presence may take different forms [42]. Any adaptation or extension of these instruments should be accompanied by renewed evidence of validity, reliability, and, where cross‐country comparisons are intended, measurement invariance [44]. Finally, greater participation of both highly productive and currently less‐connected countries in multinational research consortia could broaden the cultural basis of the evidence and strengthen the field's capacity for meaningful cross‐context comparison.

This study has some limitations. Because the analysis was conducted only with records indexed in the Web of Science Core Collection and published in English, studies in Scopus, CINAHL, and local indexes, as well as non‐English literature, may have been excluded. Citation indicators are sensitive to the data retrieval date and may change over time. In addition, keyword‐based analyses relied on Author Keywords and therefore partly reflect the terminology selected by the authors of the original publications.

## 5 Conclusion

This bibliometric analysis maps the development of caring behaviours research in nursing from 1987 to 2025. The literature shows steady growth and is distributed across a wide range of journals. However, international co‐authorship remains relatively limited **(14.25%)**, suggesting that much of the evidence is still produced within national and regional contexts. Citation structures indicate stable theoretical and measurement anchors, with Watson and Swanson remaining influential. Key measurement‐focused publications, particularly Wu (2006), also help connect multiple research clusters. The co‐citation analysis further identifies Wu (2006) as the principal bridging reference, while Wolf (1994) also occupies a prominent position within the intellectual structure of the field.

The thematic map places the broad cluster centred on caring behaviours within the basic‐themes quadrant, indicating that it remains highly relevant to the field but has comparatively lower internal development. In contrast, work environment, together with humanistic care and related terms, occupies the motor‐theme quadrant, pointing to increasing thematic development around the organizational and humanistic conditions in which caring is enacted. Measurement‐related themes show a more differentiated pattern: Reliability, validity, and instrument development appear as specialised niche themes, whereas validation is positioned in the emerging or declining quadrant and psychometric properties remain comparatively peripheral. These findings suggest that, despite the continued influence of established instruments, measurement research remains somewhat separated from the broader thematic core and may benefit from renewed validation across changing clinical and cultural contexts.

Overall, caring behaviours research in nursing appears to be an established and expanding field, supported by stable theoretical foundations and a strong measurement tradition. Future research would benefit from stronger international collaboration and more integrated multi‐country programs, particularly those using comparable methods and examining the cross‐cultural equivalence of caring‐behaviour measures. The recent prominence of empathy, emotional intelligence, compassion fatigue, and work environment also suggests that future work should consider not only how caring is perceived by patients, but also the emotional, professional, and organizational conditions that enable nurses to sustain caring behaviours. As nursing care increasingly incorporates remote and digitally mediated interactions, it may also be useful to examine whether established concepts and measurement tools adequately capture relational presence, communication, and continuity of caring in these settings. Such work could extend the field without abandoning the theoretical and measurement foundations that have shaped it over the past four decades.

## Author Contributions

İ.Ö.: Conceptualisation, Methodology, Data Curation, Formal Analysis, Visualisation, Writing – Original Draft, Writing – Review and Editing. S.T.: Methodology, Data Curation, Validation, Writing – Review and Editing.

## Funding

The authors have nothing to report.

## Conflicts of Interest

The authors declare no conflicts of interest.

## Supporting information


**File S1:** The BIBLIO checklist for reporting the bibliometric reviews of the biomedical literature.

**File S2:** Author Keyword Preprocessing and Harmonization Decisions.
**Table S1:** Synonym merging and harmonization of Author Keywords.
**Table S2:** Terms intentionally retained as distinct concepts.

**Figure S1:** Most relevant sources in the caring behaviours literature.
**Figure S2:** Core sources according to Bradford's Law.
**Figure S3:** Most productive authors in the caring behaviours literature.
**Figure S4:** Most locally cited authors in the caring behaviours literature.
**Figure S5:** Author productivity according to Lotka's Law.
**Figure S6:** Corresponding authors' countries and international collaboration patterns.
**Table S3:** Scientific production by corresponding author's country and collaboration type.

**Table S4:** Centrality Indicators of Author Keywords by Cluster.
**Table S5:** Thematic Cluster Classification Based on Callon Centrality and Density.
**Table S6:** Centrality Measures of Authors in the Co‐authorship Network.
**Table S7:** Centrality Measures of Countries in the Collaboration Network.
**Table S8:** Centrality Measures of Referenced Documents in the Co‐citation Network.

## Data Availability

The data that support the findings of this study are available on request from the corresponding author. The data are not publicly available due to privacy or ethical restrictions.
