# Supplementary material for: Caring Behaviours in Nursing: A Bibliometric Analysis of Global Research Trends and Knowledge Structure
Source: Scand J Caring Sci. 2026 Oct 1;40(4):e70318. doi: 10.1111/scs.70318 (PMC13628527; doi:10.1111/scs.70318)
Supplement: Supplementary file 1 [file SCS-40-0-s001.docx]

**Figure S1. Most relevant sources in the caring behaviours literature.**

**Figure S2. Core sources according to Bradford’s Law.**

**Figure S3. Most productive authors in the caring behaviours literature.**

**Figure S4. Most locally cited authors in the caring behaviours literature.**

**Figure S5. Author productivity according to Lotka’s Law.**

**Figure S6. Corresponding authors’ countries and international collaboration patterns.**

**Table S3. Scientific production by corresponding author’s country and collaboration type.**

| **Country** | **Articles** | **Scp** | **Mcp** | **Freq** | **Mcp_Ratio** |
| --- | --- | --- | --- | --- | --- |
| Usa | 85 | 79 | 6 | 0,24216524 | 0,07058824 |
| Iran | 45 | 41 | 4 | 0,12820513 | 0,08888889 |
| Chına | 38 | 33 | 5 | 0,10826211 | 0,13157895 |
| Turkey | 32 | 30 | 2 | 0,09116809 | 0,0625 |
| Indonesıa | 19 | 17 | 2 | 0,05413105 | 0,10526316 |
| Cyprus | 8 | 3 | 5 | 0,02279202 | 0,625 |
| Sweden | 8 | 7 | 1 | 0,02279202 | 0,125 |
| Ethıopıa | 7 | 5 | 2 | 0,01994302 | 0,28571429 |
| Italy | 6 | 4 | 2 | 0,01709402 | 0,33333333 |
| Malaysıa | 6 | 3 | 3 | 0,01709402 | 0,5 |
| Egypt | 4 | 2 | 2 | 0,011396011 | 0,5 |
| Korea | 4 | 4 | 0 | 0,011396011 | 0 |
| Slovenıa | 4 | 4 | 0 | 0,011396011 | 0 |
| Brazıl | 3 | 3 | 0 | 0,008547009 | 0 |
| Canada | 3 | 2 | 1 | 0,008547009 | 0,333333333 |
| Ecuador | 3 | 2 | 1 | 0,008547009 | 0,333333333 |
| Greece | 3 | 2 | 1 | 0,008547009 | 0,333333333 |
| Japan | 3 | 3 | 0 | 0,008547009 | 0 |
| Jordan | 3 | 2 | 1 | 0,008547009 | 0,333333333 |

SCP, single-country publication; MCP, multiple-country publication; MCP ratio, proportion of multiple-country publications.
