# Supplementary material for: Caring Behaviours in Nursing: A Bibliometric Analysis of Global Research Trends and Knowledge Structure
Source: Scand J Caring Sci. 2026 Oct 1;40(4):e70318. doi: 10.1111/scs.70318 (PMC13628527; doi:10.1111/scs.70318)
Supplement: Supplementary file 2 [file SCS-40-0-s002.docx]

| **Table S4. Centrality Indicators of Author Keywords by Cluster** | | | | |
| --- | --- | --- | --- | --- |
| **Node** | **Cluster** | **Betweenness** | **Closeness** | **PageRank** |
| Caring behaviors | 1 | 608,153474 | 0,01754386 | 0,19552198 |
| Nurses | 1 | 227,534055 | 0,01408451 | 0,10007406 |
| Caring | 1 | 102,636837 | 0,01351351 | 0,08288838 |
| Patient satisfaction | 1 | 0,07710843 | 0,01111111 | 0,01622124 |
| Perceptions | 1 | 3,99102826 | 0,01219512 | 0,03097761 |
| Emotional intelligence | 1 | 0,51266104 | 0,01149425 | 0,02109073 |
| Behavior | 1 | 1,14756611 | 0,01010101 | 0,01352467 |
| Patients | 1 | 1,4071206 | 0,01111111 | 0,01930594 |
| İntensive care unit | 1 | 0,01927711 | 0,01111111 | 0,01098363 |
| Professional values | 1 | 0,0146789 | 0,01098901 | 0,01056274 |
| Care | 1 | 0,04432133 | 0,01086957 | 0,00870237 |
| Ethical sensitivity | 1 | 0,0146789 | 0,01098901 | 0,01140968 |
| Burnout | 1 | 0,15391037 | 0,01123596 | 0,01217277 |
| Cancer | 1 | 0 | 0,00980392 | 0,00556568 |
| Compassion fatigue | 1 | 0 | 0,00980392 | 0,00556568 |
| Ethiopia | 1 | 0 | 0,01052632 | 0,00953342 |
| Nurse managers | 1 | 0 | 0,00980392 | 0,00477429 |
| Psychometrics | 1 | 0 | 0,00952381 | 0,00626472 |
| Work environment | 1 | 0 | 0,01098901 | 0,01052016 |
| Behaviors | 1 | 0 | 0,00961538 | 0,00864735 |
| Hospitals | 1 | 0 | 0,01111111 | 0,00941861 |
| Moral sensitivity | 1 | 0 | 0,00980392 | 0,00635708 |
| Validation | 1 | 0 | 0,01041667 | 0,00625755 |
| China | 1 | 0 | 0,00980392 | 0,00477429 |
| Disgust sensitivity | 1 | 0 | 0,00980392 | 0,00556568 |
| Emergency department | 1 | 0 | 0,01052632 | 0,00636425 |
| Nursing students | 2 | 56,7869589 | 0,01265823 | 0,07941498 |
| Nursing | 2 | 54,1892662 | 0,01162791 | 0,04731141 |
| Nursing care | 2 | 76,302329 | 0,01333333 | 0,05651978 |
| Nursing education | 2 | 45,4736717 | 0,01136364 | 0,03389738 |
| Empathy | 2 | 0 | 0,01052632 | 0,01703365 |
| Clinical instructors | 2 | 0 | 0,01075269 | 0,01691912 |
| Ethics | 2 | 0,38232528 | 0,00990099 | 0,01178063 |
| Students | 2 | 0,59852474 | 0,01098901 | 0,01302517 |
| Education | 2 | 0,01927711 | 0,00952381 | 0,00842567 |
| Simulation | 2 | 0,03738318 | 0,01020408 | 0,00887837 |
| Clinical learning environment | 2 | 0 | 0,01010101 | 0,00850059 |
| Quality of care | 2 | 0 | 0,01030928 | 0,00711542 |
| Care-Q | 2 | 0 | 0,01010101 | 0,00624174 |
| Covid-19 | 3 | 0,5035472 | 0,0106383 | 0,01322236 |
| Anxiety | 3 | 0 | 0,00990099 | 0,00681774 |
| Psychometric properties | 4 | 0 | 0,00763359 | 0,00551157 |
| Humanistic care | 5 | 0 | 0,00862069 | 0,00478145 |
| Instrument development | 6 | 0 | 0,0075188 | 0,00474894 |
| Nursing theory | 7 | 0 | 0,00833333 | 0,00474123 |
| Reliability | 8 | 0 | 0,00869565 | 0,01103412 |
| Validity | 9 | 0 | 0,00869565 | 0,01103412 |

**Table S5. Thematic Cluster Classification Based on Callon Centrality and Density**

| Cluster | CallonCentrality | CallonDensity | RankCentrality | RankDensity | ClusterFrequency |
| --- | --- | --- | --- | --- | --- |
| Phenomenology | 0 | 33,3333333 | 4,5 | 11,5 | 3 |
| Caring Behaviors | 1,71185236 | 23,8668089 | 16 | 6 | 466 |
| Work Environment | 0,06666667 | 40,4444444 | 10 | 16 | 18 |
| Cancer | 0,15 | 25 | 11 | 7,5 | 9 |
| Validation | 0 | 25 | 4,5 | 7,5 | 4 |
| Psychometric Properties | 0,16666667 | 20 | 13 | 2,5 | 5 |
| Nursing Students | 0,38116835 | 23,3548871 | 15 | 5 | 119 |
| Nurse Managers | 0,15666667 | 20 | 12 | 2,5 | 5 |
| Students | 0,17222222 | 21,6666667 | 14 | 4 | 11 |
| Patient Satisfaction | 0,04895833 | 6,25 | 9 | 1 | 16 |
| İnstrument Development | 0 | 33,3333333 | 4,5 | 11,5 | 7 |
| Leadership | 0 | 33,3333333 | 4,5 | 11,5 | 3 |
| Reliability | 0 | 37,5 | 4,5 | 15 | 8 |
| Survey | 0 | 33,3333333 | 4,5 | 11,5 | 3 |
| Grounded Theory | 0 | 33,3333333 | 4,5 | 11,5 | 3 |
| Oncology Nursing | 0 | 33,3333333 | 4,5 | 11,5 | 3 |

**Table S6. Centrality Measures of Authors in the Co-authorship Network**

| **Node** | **Cluster** | **Betweenness** | **Closeness** | **PageRank** |
| --- | --- | --- | --- | --- |
| Sjoden Po | 1 | 0 | 1 | 0,02702703 |
| Vonessen L | 1 | 0 | 1 | 0,02702703 |
| Kersu Ö | 2 | 0 | 1 | 0,02702703 |
| Sayilan Aa | 2 | 0 | 1 | 0,02702703 |
| Taylan S | 3 | 0 | 1 | 0,02702703 |
| Özkan İ | 3 | 0 | 1 | 0,02702703 |
| Boz İ | 4 | 1 | 0,5 | 0,03944485 |
| Teskereci G | 4 | 0 | 0,33333333 | 0,01523009 |
| Akgün M | 4 | 0 | 0,33333333 | 0,02640614 |
| Labrague Lj | 5 | 0 | 0,2 | 0,02655542 |
| Arulappan J | 5 | 0 | 0,2 | 0,02655542 |
| Papathanasiou IV | 5 | 1 | 0,25 | 0,03524078 |
| Tsaras K | 5 | 1 | 0,25 | 0,03524078 |
| Alikari V | 5 | 0 | 0,16666667 | 0,01154272 |
| Yetti K | 6 | 0 | 1 | 0,02702703 |
| Kuntarti K | 6 | 0 | 1 | 0,02702703 |
| Saiani L | 7 | 0 | 1 | 0,02702703 |
| Ambrosi E | 7 | 0 | 1 | 0,02702703 |
| Duffy JR | 8 | 0 | 0,5 | 0,02550564 |
| Lasiter S | 8 | 0 | 0,5 | 0,0300698 |
| Kostich K | 8 | 0 | 0,5 | 0,02550564 |
| Lee-Hsieh J | 9 | 0 | 0,33333333 | 0,03181214 |
| Turton MA | 9 | 0 | 0,33333333 | 0,02695526 |
| Kuo CL | 9 | 0 | 0,33333333 | 0,02704716 |
| Tseng HF | 9 | 0 | 0,33333333 | 0,02229355 |
| Papastavrou E | 10 | 0 | 0,11111111 | 0,03023883 |
| Karlou C | 10 | 0 | 0,11111111 | 0,02942259 |
| Patiraki E | 10 | 0 | 0,11111111 | 0,02942259 |
| Tsangari H | 10 | 0 | 0,11111111 | 0,03023883 |
| Efstathiou G | 10 | 0 | 0,11111111 | 0,02862742 |
| Merkouris A | 10 | 0 | 0,11111111 | 0,02377263 |
| Balogh Z | 10 | 0 | 0,11111111 | 0,02463685 |
| Jarosova D | 10 | 0 | 0,11111111 | 0,02463685 |
| Leino-Kilpi H | 10 | 0 | 0,11111111 | 0,02463685 |
| Suhonen R | 10 | 0 | 0,11111111 | 0,02463685 |
| Cruz JP | 11 | 0 | 1 | 0,02702703 |
| Inocian EP | 11 | 0 | 1 | 0,02702703 |

**Table S7. Centrality Measures of Countries in the Collaboration Network**

| Node | Cluster | Betweenness | Closeness | PageRank |
| --- | --- | --- | --- | --- |
| Saudi Arabia | 1 | 92,16820571 | 0,013888889 | 0,043038065 |
| Philippines | 1 | 9,217204301 | 0,010638298 | 0,029608739 |
| Egypt | 1 | 0 | 0,009803922 | 0,018149565 |
| Kazakhstan | 1 | 0 | 0,010204082 | 0,020804269 |
| Bahrain | 1 | 0 | 0,009803922 | 0,013492797 |
| Spain | 2 | 89 | 0,011494253 | 0,029992936 |
| Chile | 2 | 0 | 0,008547009 | 0,024598184 |
| Belgium | 2 | 0 | 0,008547009 | 0,024598184 |
| Ecuador | 2 | 0 | 0,008474576 | 0,010659213 |
| Usa | 3 | 327,7027342 | 0,019607843 | 0,109184937 |
| Turkey | 3 | 0 | 0,012658228 | 0,014371992 |
| Jordan | 3 | 0 | 0,012195122 | 0,007600257 |
| Oman | 3 | 6,911230287 | 0,014285714 | 0,036090434 |
| Malawi | 3 | 0 | 0,012195122 | 0,007600257 |
| Nigeria | 3 | 0 | 0,013333333 | 0,025370587 |
| İndia | 3 | 0 | 0,013333333 | 0,016188804 |
| İreland | 3 | 0 | 0,012195122 | 0,007600257 |
| Sweden | 4 | 31 | 0,0125 | 0,021690895 |
| Ethiopia | 4 | 0 | 0,009009009 | 0,016577222 |
| China | 5 | 38,79218481 | 0,013333333 | 0,038027805 |
| İndonesia | 5 | 29,74283529 | 0,013513514 | 0,024163953 |
| Malaysia | 5 | 0,6 | 0,009708738 | 0,023885249 |
| Thailand | 5 | 0,496774194 | 0,01010101 | 0,01664865 |
| İtaly | 6 | 133,4942544 | 0,016129032 | 0,044601023 |
| Greece | 6 | 77,90274774 | 0,015873016 | 0,074639774 |
| Cyprus | 6 | 9,751987718 | 0,014705882 | 0,045746276 |
| Finland | 6 | 0 | 0,011904762 | 0,037570233 |
| Czech Republic | 6 | 0 | 0,011904762 | 0,037570233 |
| Hungary | 6 | 0 | 0,011904762 | 0,037570233 |
| İran | 7 | 24,18881674 | 0,014705882 | 0,032538278 |
| Canada | 7 | 1,202020202 | 0,011363636 | 0,017734896 |
| United Kingdom | 7 | 2,579004329 | 0,013513514 | 0,020723453 |
| Pakistan | 7 | 1,25 | 0,011363636 | 0,014219491 |
| Japan | 8 | 0 | 1 | 0,028571429 |
| Australia | 8 | 0 | 1 | 0,028571429 |

**Table S8. Centrality Measures of Referenced Documents in the Co-citation Network**

| Node | Cluster | Betweenness | Closeness | PageRank |
| --- | --- | --- | --- | --- |
| Wu Y 2006 | 1 | 373,442821 | 0,00934579 | 0,06582597 |
| Wolf Z 1994 | 1 | 268,40023 | 0,00900901 | 0,05424489 |
| Papastavrou E 2011 | 1 | 107,259712 | 0,00775194 | 0,0419854 |
| Cronin SN1988 | 1 | 15,5202918 | 0,00628931 | 0,03469068 |
| Swanson KM 1991 | 1 | 11,5206629 | 0,00564972 | 0,0185032 |
| Wolf Z 1998 | 1 | 16,9235299 | 0,00680272 | 0,02571334 |
| Swanson K 1993 | 1 | 0,13114754 | 0,00324675 | 0,01437284 |
| Palese A 2011 | 1 | 47,1431407 | 0,00680272 | 0,01882708 |
| Watson J. 1988 | 1 | 2,38952068 | 0,00540541 | 0,01032291 |
| Watson J. 2008-1 | 1 | 49,3952578 | 0,00719424 | 0,02709607 |
| Watson Jean 2009 | 1 | 2,93466585 | 0,00645161 | 0,0146161 |
| Watson J. 2008-2 | 1 | 0 | 0,00327869 | 0,0168558 |
| Brilowski Ga 2005 | 1 | 16,0971259 | 0,00680272 | 0,02158659 |
| Finfgeld-Connett D 2008 | 1 | 1,54979662 | 0,00346021 | 0,02511112 |
| Omari Fh 2013 | 1 | 18,2476125 | 0,00653595 | 0,01911736 |
| Larson P 1984 | 1 | 0,42728676 | 0,00591716 | 0,01051208 |
| Zamanzadeh V 2010 | 1 | 13,2782874 | 0,00699301 | 0,02144512 |
| Baldursdottir G 2002 | 1 | 0,46055199 | 0,00598802 | 0,01674622 |
| Beck Ct 1999 | 1 | 2,74304374 | 0,00564972 | 0,01546476 |
| O'connell Elizabeth 2008 | 1 | 0,57816894 | 0,00591716 | 0,01577186 |
| Papastavrou E 2012 | 1 | 0,48511036 | 0,00584795 | 0,01806576 |
| Watson J. 1979 | 1 | 2,06952068 | 0,00534759 | 0,010183 |
| Wolf Zane 2003 | 1 | 13,606425 | 0,00689655 | 0,01586599 |
| Liu Je 2006 | 1 | 0 | 0,00334448 | 0,02108417 |
| Von Essen 2003 | 1 | 5,77350817 | 0,00628931 | 0,01726425 |
| Watson Jean 2007 | 1 | 0 | 0,00298507 | 0,00733394 |
| Boykin A. 2001 | 1 | 0 | 0,00287356 | 0,01162731 |
| Chang Y 2005 | 1 | 3,21042957 | 0,00613497 | 0,0196948 |
| Karlou C 2015 | 1 | 2,41215217 | 0,00621118 | 0,01218982 |
| Labrague LJ 2015 | 2 | 67,5145632 | 0,00775194 | 0,03309036 |
| Labrague LJ 2017 | 2 | 44,012713 | 0,00729927 | 0,03460216 |
| Kursun S 2012 | 2 | 6,03232734 | 0,00671141 | 0,01116423 |
| Oluma A 2020 | 2 | 106,235381 | 0,00787402 | 0,01821284 |
| Sarafis P 2016 | 2 | 118,080342 | 0,008 | 0,01607909 |
| Zamanzadeh Vahid 2014 | 2 | 14,5295244 | 0,00645161 | 0,0317052 |
| Burtson PL 2010 | 2 | 32,0006131 | 0,00699301 | 0,01560813 |
| Khademlan Z 2008 | 2 | 233,545925 | 0,00826446 | 0,02209197 |
| Murphy F 2009 | 2 | 8,27318077 | 0,0070922 | 0,02107341 |
| Wade GH 2006 | 2 | 0,69020898 | 0,00578035 | 0,01384027 |
| Beck CT 2001 | 2 | 121,877509 | 0,0070922 | 0,01406818 |
| Taylan S 2021 | 2 | 0 | 0,00645161 | 0,01078078 |
| Alikari V 2023 | 2 | 22,0121729 | 0,0075188 | 0,01404121 |
| Aupia A 2018 | 2 | 15,1943519 | 0,00740741 | 0,01860921 |
| Watson J. 2018 | 2 | 0,53860066 | 0,00671141 | 0,01127605 |
| Gozutok Konuk 2019 | 2 | 0 | 0,00645161 | 0,01594824 |
| Karaöz S 2005 | 2 | 59,6210392 | 0,00740741 | 0,02246512 |
| Loke Jcf 2015 | 2 | 0,10772013 | 0,00671141 | 0,01594449 |
| Watson J. 2002 | 2 | 20,1158598 | 0,00653595 | 0,00803972 |
| Watson R 1999 | 2 | 13,6179673 | 0,0070922 | 0,01695704 |
| Hosseinzadeh H. 2019 | 2 | 0 | 0,00564972 | 0,01228388 |
