# Supplementary material for: Caring Behaviours in Nursing: A Bibliometric Analysis of Global Research Trends and Knowledge Structure
Source: Scand J Caring Sci. 2026 Oct 1;40(4):e70318. doi: 10.1111/scs.70318 (PMC13628527; doi:10.1111/scs.70318)
Supplement: Supplementary file 3 [file SCS-40-0-s003.docx]

**Supplementary File 2. Author Keyword Preprocessing and Harmonisation Decisions**

Before keyword-based analyses, Author Keywords (DE) were manually reviewed to reduce variation arising from singular/plural forms, spelling differences, punctuation, and semantically equivalent expressions. Harmonisation was performed conservatively to avoid unnecessary alteration of the conceptual structure.

**Table S1. Synonym merging and harmonisation of Author Keywords**

| **Standardised Author Keyword** | **Original variants merged** | **Type of harmonisation** |
| --- | --- | --- |
| caring behaviors | caring behavior; caring behaviours; caring behaviour; care behaviors; care behavior; caring behavour; nurse caring behavior; nurse-caring behavior; nurses caring behavior; nursing caring behavior; nursing caring behaviors; nurses' care behaviour | Spelling, singular/plural, punctuation, and terminological harmonisation |
| nurses | nurse; nurses. | Singular/plural and punctuation harmonisation |
| nursing students | nursing student; student nurses | Singular/plural and terminological harmonisation |
| clinical instructors | clinical instructor | Singular/plural harmonisation |
| patients | patient | Singular/plural harmonisation |
| perceptions | perception | Singular/plural harmonisation |
| intensive care unit | intensive care units | Singular/plural harmonisation |
| nurse managers | nurse manager | Singular/plural harmonisation |
| professional values | professional value | Singular/plural harmonisation |
| nursing education | nurse education | Terminological harmonisation |

**Table S2. Terms intentionally retained as distinct concepts**

| **Terms retained separately** | **Rationale** |
| --- | --- |
| caring behaviors / caring | These terms were retained separately because *caring behaviors* represents observable or measurable expressions of caring, whereas *caring* refers to the broader theoretical concept. |
| nurses / nursing | *Nurses* refers to the professional group, whereas *nursing* refers to the discipline or professional field. |
| caring behaviors / nursing care | *Nursing care* was retained separately because it represents a broader concept of care delivery. |
| reliability / validity / validation / psychometric properties / instrument development | These terms were retained separately because they represent related but analytically distinct aspects of measurement research. |

**No additional remove list was applied. Keyword preprocessing was restricted to synonym merging, spelling and punctuation harmonisation, and singular/plural harmonisation as reported above.**
